# Case report: SAF-189s is a potent inhibitor in a lorlatinib-resistant NSCLC patient with acquired compound mutations ALK L1196M and D1203N

**DOI:** 10.3389/fphar.2023.1197163

**Published:** 2023-12-11

**Authors:** Na Li, Huihui Li, Ding Wang, Xiaoling Xu

**Affiliations:** ^1^ Department of Medical Oncology, Shaoxing Second Hospital, Shaoxing, Zhejiang, China; ^2^ The Second Clinical Medical College, Wenzhou Medical University, Wenzhou, Zhejiang, China; ^3^ Zhejiang Key Laboratory of Diagnosis & Treatment Technology on Thoracic Oncology (Lung and Esophagus), The Cancer Hospital of the University of Chinese Academy of Sciences (Zhejiang Cancer Hospital), Institute of Basic Medicine and Cancer (IBMC), Chinese Academy of Sciences, Hangzhou, Zhejiang, China; ^4^ Department of Radiation Oncology, Shanghai Pulmonary Hospital, Tongji University School of Medicine, Shanghai, China

**Keywords:** SAF-189s, ALK L1196M, ALK D1203N, lorlatinib resistance, treatment

## Abstract

Acquired anaplastic lymphoma kinase (ALK) mutation is the major resistant mechanism to ALK tyrosine kinase inhibitors (TKIs) in non-small cell lung cancer (NSCLC) patients. At present, treatment options after acquiring secondary ALK mutations are still limited. Here, we report on a patient with metastatic ALK-rearranged NSCLC who was sequentially treated with ALK TKIs, from crizotinib to lorlatinib, and developed rare acquired compound ALK mutations (L1196M and D1203N) that confer resistance to lorlatinib. Moreover, our report describes the clinical response of an NSCLC patient with these compound mutations to multiple anti-tumor therapies. Among them, the patient was treated with SAF-189s 120 mg daily and had a stable disease lasting 3 months. Chemotherapy (pemetrexed-carboplatin) combined with bevacizumab was then administered. She achieved a partial response, which was maintained for 7 months as the best response. Since both SAF-189s and chemotherapy have shown a clear antitumor effect, they may be viable therapeutic options for these patients. Thus, our study can provide some reference in the treatment of NSCLC patients with ALK L1196M/D1203N compound mutations.

## Introduction

Lung cancer remains the leading cause of cancer-related mortality worldwide, with a less than 20% 5-year survival rate (Siegel et al., 2020). Non-small cell lung cancer (NSCLC) is the major histological subtype of lung cancer, of which 3%–7% of cases harbor oncogenic anaplastic lymphoma kinase (ALK) gene rearrangements (Tsao et al., 2016). Lorlatinib, a highly potent third-generation ALK tyrosine kinase inhibitor (TKI), can effectively overcome most secondary resistance mutations caused by first/second-generation ALK-TKIs, and possesses high blood-barrier penetration by reducing the P-glycoprotein-dependent efflux (Bauer et al., 2020; Naito et al., 2021). Unfortunately, similar to other ALK-TKIs, acquired resistance to lorlatinib is also inevitable. The major resistance mechanisms are the acquisition of two or more ALK secondary mutations, gene amplification, bypass signaling activation (such as EGFR, Met, KRAS, and c-KIT), and histological and/or phenotypical changes (such as small cell lung cancer transformation and EMT) (Gainor et al., 2016; Yanagitani et al., 2020; Remon et al., 2021).

Treatment-refractory compound ALK mutations are more common in NSCLC patients resistant to lorlatinib, and the most frequent combinations are G1202R/L1196M and D1203N/1171N (Dagogo-Jack et al., 2019). Recent studies have reported that the ALK L1196M/D1203N compound mutations confer high resistance to lorlatinb; however, the mechanism of acquired resistance and the therapeutic strategy for lorlatinib-relapsed patients with these novel compound mutations remain to be elucidated (Recondo et al., 2020; Hua et al., 2022; Katayama et al., 2023). Here, we report rare acquired ALK compound mutations (ALK L1196M and D1203N) conferring resistance to lorlatinib, and we are the first to describe the clinical responses of an NSCLC patient with these compound mutations to multiple anti-tumor therapies. Among them, SAF-189s and chemotherapy showed a clear antitumor effect.

## Case presentation

A 29-year-old young woman was diagnosed with left lung adenocarcinoma and bone metastases (cT1N3M1, IV). She had received two cycles of chemotherapy consisting of pemetrexed and carboplatin from August 2016 to October 2016 at another hospital. Her Eastern Cooperative Oncology Group performance score (ECOG-PS) was 1. She came to our clinic for the following therapy. Before treatment, a biopsy of the enlarged cervical lymph nodes was performed, and next-generation sequencing (NGS) identified the rearrangement of ALK gene and the mutation of TP53 gene in the biopsy specimen. Thus, she started crizotinib 250 mg twice daily and had confirmed partial response (PR) as the best response. After 30 months, the patient presented with progression of metastases in the sternum, vertebrae, ribs, and ilium. Plasma NGS revealed the emergence of ALK D1203N. Then, the third-generation ALK inhibitor lorlatinib was administered at a dose of 100 mg daily from May 2019 and she also achieved PR. However, a new liver metastasis and enlarged abdominal and inguinal lymph nodes were detected at a regular CT scan in November 2020, while pulmonary lesions were well controlled. Ultrasound-guided biopsy of the left inguinal lymph node confirmed that the nature of the lesion was lung adenocarcinoma metastasis, and NGS of the biopsy specimen revealed two acquired mutations, ALK L1196M (mutation allelic frequency (MAF): 14.62%) and ALK D1203N (MAF: 13.22%). Thus, progressive disease (PD) was confirmed and the patient reached a PFS of 19 months to lorlatinib treatment.

The patient was then enrolled into a phase I/II trial of SAF-189s (ClinicalTrials.gov identifier: NCT04237805), and was treated with SAF-189s 120 mg daily in December 2020. After 6 weeks of treatment, the first restaging CT scan showed a stable disease (SD) based on RECIST 1.1. NGS of a liver biopsy specimen was also performed and revealed the persistence of the same acquired ALK mutations. However, in March 2021, a CT scan showed worsening liver metastases and new supraclavicular lymphadenopathy, suggesting PD. The response was maintained for 3 months. The toxicities of SAF-189s for the patient were tolerable, with only grade 3 hypercholesterolemia. Her ECOG-PS was 2 throughout the treatment period. After progression with SAF-189s therapy, NGS of a plasma and supraclavicular lymph node biopsy specimen revealed the continual persistence of ALK L1196M and ALK D1203N.

**FIGURE 1:**
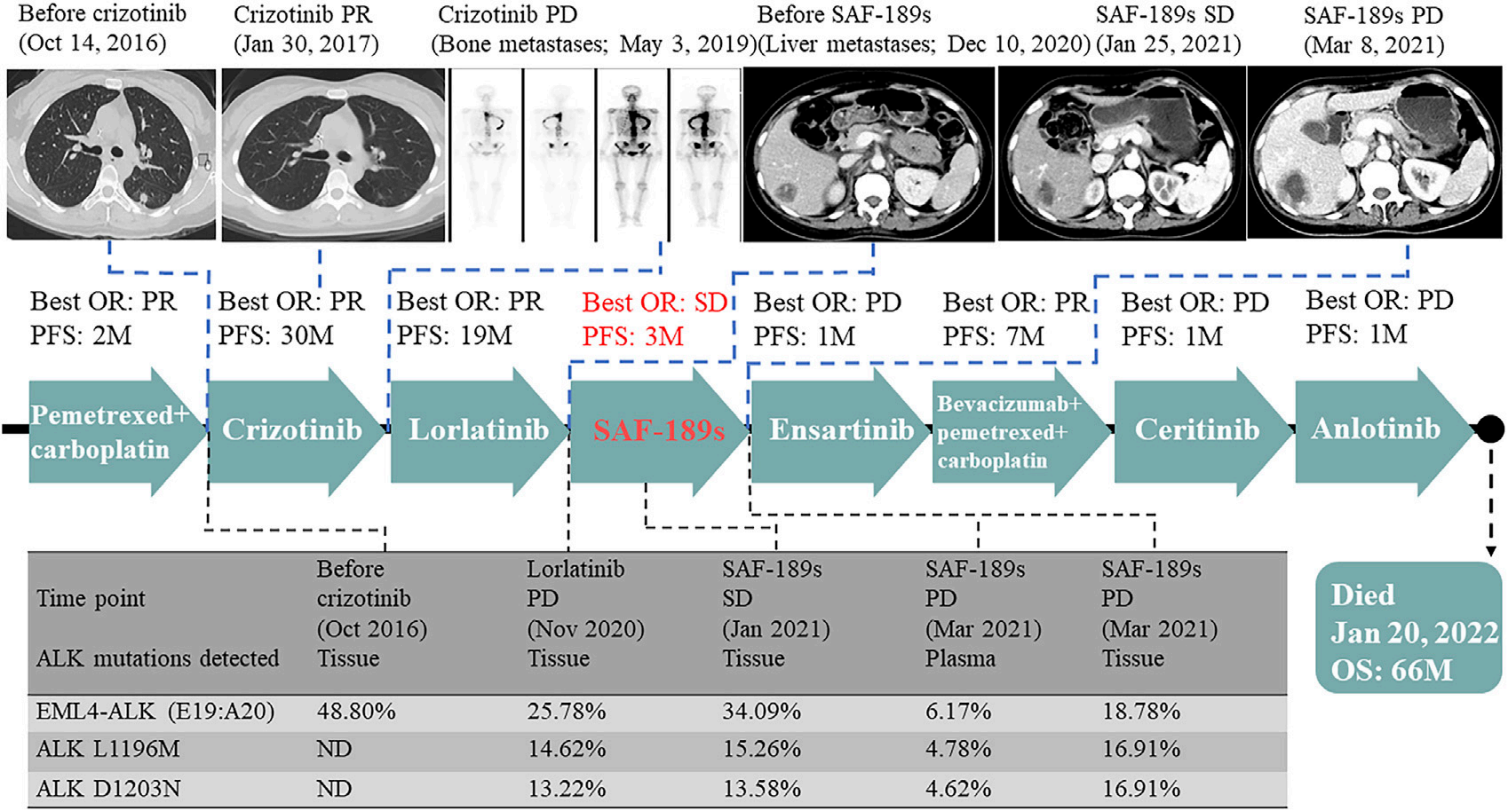
Illustrated summary of the treatment received by the patient, including best response and progression-free survival. Mutations and their corresponding allelic fractions detected by NGS are also presented at the bottom of the figure.

**FIGURE 2:**
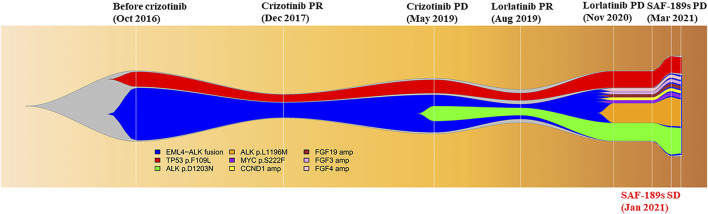
Visualizing the tumor gene evolution of the patient with a fishplot.

The therapy was then switched to ensartinib 225 mg daily. A brain MRI in April 2021 revealed metastases in the right frontal and parietal lobes, which indicated that the disease had progressed again, with a PFS of only 1 month. After whole-brain radiotherapy (30 Gy/10 F), chemotherapy (pemetrexed-carboplatin) combined with bevacizumab was administered, she achieved PR, and lasted for 7 months. After the cancer relapsed, she received another two therapies, ceritinib and anlotinib, but responded to neither. Later on, the patient become increasingly frail and died on 20 January 2022, with an overall survival of 66 months (Figures 1, 2).

## Discussion

Sequential treatment of ALK-TKIs could lead to the stepwise accumulation of mutations mediating the high resistance to ALK inhibitors (Yoda et al., 2018). Our patient was sequentially treated with first-generation (crizotinib) and third-generation (lorlatinib) ALK inhibitors, and acquired ALK compound mutations (L1196M and D1203N) conferring high-level resistance to lorlatinib. In post-lorlatinib progressed patients, ≥2 ALK mutations can be detected in 48% of plasma specimens, the most frequent combinations being ALK G1202R/L1196M and ALK D1203N/1171N (Dagogo-Jack et al., 2019). Some compound mutations, such as ALK C1156Y/L1198F and ALK I1171N/L1256F, are resistant to lorlatinib but re-sensitize to crizotinib and alectinib, suggesting that patients with these mutations can be re-treated with first- or second-generation ALK-TKIs. However, compound mutations such as ALK G1202R/L1196M confer high resistance to all ALK-TKIs (Mizuta et al., 2021). Thus, the identification of compound ALK mutations is important for developing therapeutic strategies and predicting prognosis. At present, only a few studies have reported ALK L1196M/D1203N compound mutations in lorlatinib-resistant patients, while there is no relevant report on the subsequent therapeutic strategy.

After lorlatinib resistance, our patient was first enrolled into a phase I/II trial of SAF-189s and treated with SAF-189s 120 mg daily. The best response was SD and the PFS was 3 months. The adverse event of SAF-189s was hypercholesterolemia, indicating that the lipid profile should be tested periodically during the treatment. Moreover, she also had a clinical response to the chemotherapy treatment (pemetrexed-carboplatin) and bevacizumab, achieving a PFS of 7 months with a best response of PR. SAF-189s is a new-generation ALK inhibitor that can overcome various drug-resistant mutations. In the multicenter phase I/II trial of SAF-189s, 47.6% of crizotinib-/ceritinib-resistant patients achieved PR, showing a significant antitumor effect (Xia et al., 2021; Ma et al., 2022). In this case, the patient also exhibited a clinical response to SAF-189s. However, due to the large tumor burden at the late stage of the disease, she could not achieve a satisfactory PFS. Given the effectiveness of both SAF-189s and chemotherapy, we hypothesize that combining these therapies might yield greater survival benefits, which could be a viable therapeutic option for patients with ALK L1196M and D1203N compound mutations. Overall, our report can provide guidance and reference in the treatment of patient with this acquired compound mutations in the future.

The acquisition of intra-tyrosine kinase secondary mutations is the main mechanism of resistance to ALK TKIs (Remon et al., 2021). Therefore, it is critical to dynamically monitor tumor genomic evolution during the targeted therapy. NGS is a feasible way to detect molecular profiling, and was recommended by the European Society for Medical Oncology (ESMO) in routine clinical practice for advanced NSCLC patients in 2020 (Mosele et al., 2020). With the routine use of NGS analysis, we can better understand the molecular mechanisms of resistance and formulate more effective and reasonable therapeutic strategies with ALK TKIs, particularly when administered sequentially.

## Conclusion

In conclusion, we report a metastatic ALK-rearranged NSCLC patient who was treated with sequential ALK TKIs and developed rare acquired ALK compound mutations (L1196M and D1203N) resulting in resistance to lorlatinib. Moreover, we are the first to describe the clinical responses of a patient with these ALK compound mutations to multiple therapies. Among them, SAF-189s and chemotherapy showed a clear antitumor effect, which may be viable therapeutic options. Thus, our study can provide some reference in the treatment of patients with ALK L1196M/D1203N compound mutations in the future.

## Data Availability

The original contributions presented in the study are included in the article/Supplementary Material, further inquiries can be directed to the corresponding author.
